# Fatal Methaemoglobin Intoxication Following Ketamine Infusion in a Depressed MELAS Patient: A Possible Association?

**DOI:** 10.1155/crcc/5200754

**Published:** 2025-11-29

**Authors:** Josef Finsterer

**Affiliations:** ^1^ Neurology Department, Neurology & Neurophysiology Center, Vienna, Austria

**Keywords:** ketamine, m.3243A>G, MELAS, methemoglobin, sudden unexplained death in epilepsy

## Abstract

Methemoglobinemia is defined as an increase in methemoglobin of > 2% of hemoglobin. Ketamine is increasingly used for severe depression. To our knowledge, a patient with mitochondrial encephalopathy, lactic acidosis and stroke‐like episodes (MELAS) who died suddenly and unexpectedly 1–12 h after ketamine infusion with high postmortem methemoglobin has not yet been reported. The patient was a 32‐year‐old woman with MELAS, manifesting as migraine, deafness, diabetes, bloating, and a seizure, due to the mtDNA variant m.3243A>G who received a single infusion of ketamine for the treatment of depression and posttraumatic stress disorder. Presumably, 1–12 h later, the patient died in her apartment, initially with no apparent cause for her demise. The autopsy revealed a methemoglobin level of 71% (normal: 0‐2%). Whether ketamine was responsible for the death remains speculative, but ketamine has a mitochondrial toxic effect by reducing ATP production and impairing methemoglobin reductase, which may have led to methemoglobinemia. In conclusion, ketamine should be administered with caution in MELAS patients, particularly those with a history of seizures, and if unavoidable, these patients should be carefully monitored during and after ketamine infusion.

## 1. Introduction

Methemoglobin is a hemoglobin derivative in which the normally divalent central iron atom is oxidized to trivalent iron. It belongs to the so‐called dyshemoglobins [1]. Methemoglobinemia is defined as an increase in methemoglobin of > 2% of hemoglobin [1]. Signs of hypoxia (e.g., headache, drowsiness, fatigue, weakness, nausea, and mild cyanosis [despite normal O_2_ saturation]) occur from a methemoglobin value of 15–20% of total hemoglobin [1]. At a level of 40–50%, severe cyanosis, confusion, and respiratory distress occur, and from a methaemoglobin level of 60–70%, coma and ultimately death can occur [1]. Methemoglobinemia can be primary (genetic) or acquired (secondary) [1]. Primary methemoglobinemia is due to an inherited methemoglobin reductase deficiency or an inherited glucose‐6‐phosphate dehydrogenase deficiency [1]. Secondary methemoglobinemia can be caused by intoxication (aniline, amyl nitrate, antipyrine, chlorate, insecticides, Na‐nitroprusside, nitrates, nitrobenzene, phenacetin, sulfamethoxazole), drugs (local anesthetics [lidocaine, benzocaine, prilocaine, procaine], primaquine, sulfonamides, nitroglycerin, phenacetin, paracetamol), or illegal drugs (e.g., poppers) [1].

Ketamine is a phencyclidine derivative, an antagonist of the Ca^2+^‐permeable N‐methyl‐d‐aspartate (NMDA) receptors of the glutamate type. Ketamine is a drug from the class of general anesthetics. Due to its anesthetic, analgesic, and psychotropic effects, it is used to induce and maintain anesthesia, but also as an analgesic in emergency medicine [2]. Ketamine can also be administered for severe depression [3]. In recent years, it has been increasingly abused as an illegal drug and party drug [4]. A patient with MELAS who died suddenly and unexpectedly from methaemoglobin intoxication after a ketamine infusion is not known.

## 2. Case Report

The patient is a 32‐year‐old woman with MELAS due to the m.3243A>G variant, which phenotypically manifested as severe migraine, deafness, diabetes, bloating, and a seizure. Her current medication included tryptans, propranolol, and ondansetron. She died unexpectedly, presumably 1–12 h after a single, first‐time ketamine infusion (0.5 mg/kg over 40 min) [5] in the private practice of a psychiatrist for the treatment of depression and post‐traumatic stress disorder (following a seizure). No cause of sudden death was found at autopsy, but the morgue detected a methemoglobin level of 71% (normal 0–2% of hemoglobin levels) (Table 1). The postmortem toxicological examination also included tests for alcohol, acetone, amphetamines, isopropanol, methanol, benzodiazepines, buprenorphine, cannabinoids, carisoprodol, cocaine metabolites, fentanyl, methadone, methamphetamine, opiates, oxycodone, phencyclidine, and zolpidem, which were all negative (Table 1). The ketamine blood level was 0.16 mg/L, and the propranolol blood level was 11 mg/L (normal) (Table 1). MELAS was originally diagnosed in her 62‐year‐old mother, who was a carrier of the m.3243A>G variant with a heteroplasmy rate of 31% in saliva and 18% in blood. She phenotypically showed migraine, hypoacusis, a distended abdomen, diabetes, muscle weakness, extreme fatigue, multiple allergies, and a stroke‐like attack at the age of 50. Serum lactate was normal and her methemoglobin was 0.8%.

**Table 1:** Summary of autopsy findings of the index patient.

External description
Green emesis in the hair
Tache noire
Drying artifact in the lips and fingertips
Rigor mortis
Putrefactive discoloration over the lower abdomen
Early vascular marbling along the lower neck
Remainder unremarkable
External and internal injuries
None
Internal examination
Unremarkable
Microscopic examination
Lungs (vessels congested, variable amounts of edema in airspaces)
Liver (postmortem gas artifact)
Pancreas (diffuse autolysis)
Remained: unremarkable including the brain
Toxicology
Drugs of abuse screen (ELISA): all negative
Amphetamines: all negative
Ketamine: 0.16 mg/dL
Norketamine: positive
Propranolol 11 mg/L
Methemoglobin 71%
Vitreous chemistry
Chloride 112 mmol/L
Creatinine 1.2 mg/dL
Glucose 22 mg/dL
Potassium 29.6 mmol/L
Sodium 127 mmol/L
Urea nitrogen 21 mg/dL

## 3. Discussion

The cause of the index patient’s sudden unexplained death remains unknown, but several speculations are warranted. Presumably, the index patient died of methemoglobin intoxication, but the cause of the excessively high postmortem methemoglobin levels remains speculative. Primary methemoglobinemia was ruled out because no hereditary methemoglobinemia was found in the family history and the mother also had normal serum methemoglobin levels. Secondary methemoglobinemia was also ruled out as postmortem toxicology testing did not reveal any of the substances known to cause methemoglobinemia [1]. The patient had not received any local anesthetics, antibiotics, antimalarials, nitroglycerin, or nitroprusside shortly before her death. Neither the medical history nor the autopsy revealed any evidence of poisoning with nitrates, nitrites, aniline dyes, nitrobenzene, metoclopramide, phenazopyridine, herbicides, or pesticides. Whether the ketamine infusion played a causative role also remains speculative, as ketamine is not known to cause methemoglobinemia. It is also conceivable that the cause of death was a cardiac, pulmonary or central nervous system complication or intoxication and that the methemoglobinemia was a secondary phenomenon due to oxidative stress. Oxidative stress, regardless of its cause, may be more pronounced in patients with a genetic defect in oxidative metabolism than in patients without such a defect. It must also be considered that the methemoglobinemia was artificial.

The exact latency between the ketamine infusion and death remains unclear. However, assuming a half‐life of 3 h, death must have occurred no later than 12 h after the infusion, as ketamine was still detectable in the postmortem blood. What the patient did during this period—whether she met with anyone or took other medications—remains speculative. Assuming a half‐life of 15 min, death could have occurred the earliest 1 h after the infusion. Why death did not occur immediately after or during the infusion is also unclear. However, it is possible that secondary mechanisms (e.g., delayed norketamine metabolism, genetic reductase deficiency, persistent hypoxia) could explain the time gap. It is also conceivable that a delayed seizure occurred which was complicated by sudden unexplained death in epilepsy (SUDEP).

Ketamine is widely used for procedural sedation, treatment of acute pain, refractory status epilepticus, treatment‐resistant depression, and schizophrenia [6]. Ketamine is also increasingly used as an illicit drug [7]. It has a rapid onset of action, a relatively short half‐life (15 min to 3 h) and no respiratory depression [8]. The known side effects of ketamine are hallucinations, confusion, dizziness, nausea, high blood pressure, tachycardia, increased body temperature, and vomiting [6, 9]. No interactions between ketamine and ondansetron, ketamine and tryptanes, and ketamine and propranolol have been reported. Seizures are not a common side effect of ketamine in patients without a history of seizures, but have occasionally been reported [6, 8–10]. Arguments in favor of ketamine having caused seizures and SUDEP in the index patient is the fact that the patient had a previous seizure and that the incidence of seizures from illicit ketamine increased by 350% from 2017 to 2022 in a survey by drug regulatory agencies in the United States [11]. Another argument for a causal link between ketamine and seizures is that a correlation between the excretion of ketamine/norketamine and the frequency of seizures was found in a wastewater analysis [12].

Whether ketamine is particularly toxic to patients with mitochondrial dysfunction is not known, but there is some evidence that ketamine may impair mitochondrial function. It is known from an animal study that ketamine impairs complex I activity in the rat brain [13]. In an experimental model using cultured neurons from human induced pluripotent stem cells (iPSCs), ketamine was shown to cause mitochondrial dysfunction [14]. The major metabolite of ketamine, norketamine, has been shown to induce mitochondrial‐dependent and ER stress‐induced apoptotic death in urothelial cells via a Ca^2+^‐regulated ERK1/2 activation pathway [15]. In zebrafish, there is also evidence that ketamine can cause ATP deficiency by downregulating the atp5*α*1 subunit of ATP synthase [16]. There is also evidence that ketamine induces mitochondrial apoptosis in lymphocytes and neurons [17]. These findings suggest that the mitochondrial toxicity of ketamine may be particularly toxic in patients with a pre‐existing mtDNA defect. It is not known whether the index patient received drugs in addition to ketamine, but local anesthetics are also known to induce methemoglobinemia [18].

## 4. Conclusion

In summary, this case suggests that fatal methemoglobinemia in a MELAS patient may be associated ketamine infusion possibly due to its documented mitochondrial toxic effect. Ketamine reduces ATP production and thus impairs the function of methemoglobin reductase, leading to methemoglobinemia. Ketamine should be administered with caution in MELAS patients, particularly those with a history of seizures, and if unavoidable, these patients should be carefully monitored during and after ketamine infusions.

## Ethics Statement

The study was reviewed and approved by the IRB.

## Consent

Consent was obtained from the mother of the patient.

## Disclosure

All authors agree to be accountable for the content and conclusions of the article.

## Conflicts of Interest

The authors declare no conflicts of interest.

## Funding

No funding was received for this manuscript.

## Data Availability

The data that support the findings of this study are available from the corresponding author upon reasonable request.
